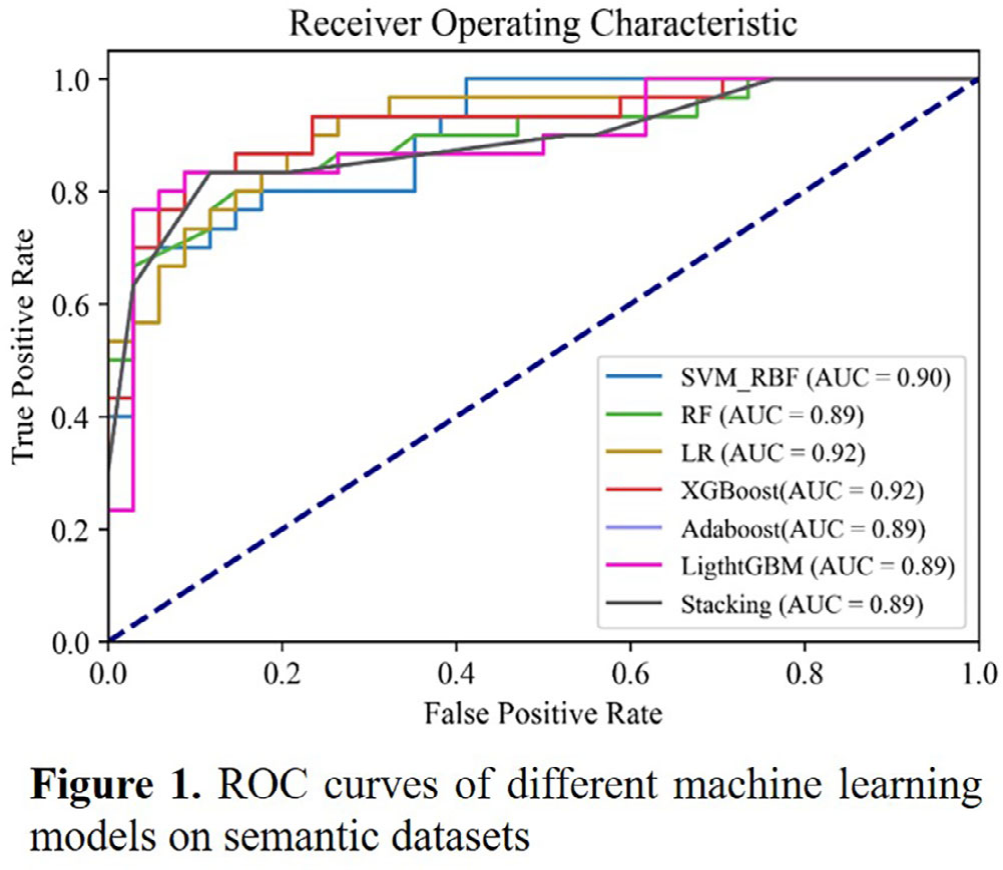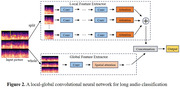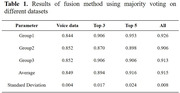# Multi‐dimensional Feature Extraction Technology of Spontaneous Speech Based on Machine Learning: Early Identification of Alzheimer's Disease

**DOI:** 10.1002/alz.090076

**Published:** 2025-01-09

**Authors:** Quan Chen, Yatian Li, Ying Wang, Yiming Li, Jingyi Gao, Nan Chen, Jingnan Wu, Ran Li

**Affiliations:** ^1^ School of Optical‐Electrical and Computer Engineering, University of Shanghai for Science and Technology, Shanghai China; ^2^ Shanghai Bestcovered Limited, Shanghai China; ^3^ College of Medical Instruments, Shanghai University of Medicine and Health Sciences, Shanghai China

## Abstract

**Background:**

Speech variations appear at early stages of Alzheimer's disease (AD) and are potential early indicators. However, the commonly used neuropsychological assessment scales as screening tools for cognitive impairments have limitations due to possible subjective bias of the assessors and the lack of sensitivity in voice detection. We propose a solution based on machine learning for multi‐dimensional feature extraction and data fusion of spontaneous speech signals.

**Method:**

Participates with gold standard diagnosis including cognitively normal, mild cognitive impairment (MCI), and mild AD underwent a speech‐based cognitive assessment (Shanghai Cognitive Screening, SCS), including picture learning and recall tasks. Their voice data were generated and created into semantic datasets and audio datasets, respectively. Then, to obtain semantic and acoustic features, the two datasets were trained in multiple machine learning models and tested separately. Finally, to separating cognitively normal and impaired (MCI+AD) participants, multi‐dimensional features of the speech signal are combined through data fusion using voting style.

**Result:**

The accuracy of this model is 91.5 ± 0.8%, with a specificity of 97.0%. In addition, there are significant differences between cognitively impaired patients and normal controls regarding instant recall as opposed to delayed recall tasks, with the most notable difference being the duration of silence in delayed recall tasks.

**Conclusion:**

This method maximizes the exploration of feature information in speech signals, greatly improving the accuracy of identification for Alzheimer's disease.